# Defining a need for rapid response and practical guidance for recurrent and metastatic squamous cell carcinoma of the head and neck (R/M SCCHN) management in France: A Delphi consensus

**DOI:** 10.1371/journal.pone.0332413

**Published:** 2025-09-19

**Authors:** Florian Clatot, Caroline Even, Amaury Daste, Christian Borel, Jérôme Fayette, Myriam Chebbah, Solenn Le Clanche, Esma Saada

**Affiliations:** 1 Centre Henri-Becquerel, Rouen, France; 2 Gustave Roussy, Villejuif, France; 3 CHU de Bordeaux, Bordeaux, France; 4 ICANS Strasbourg, Strasbourg, France; 5 Centre Léon Bérard, Lyon, France; 6 Public Health Expertise, Paris, France; 7 Medical Oncology Department, Centre Antoine Lacassagne, Nice, France; 8 Laboratory of Translational Research in Oncology, Université Côte d’Azur, Nice, France; SRM-RI: SRM Institute of Science and Technology (Deemed to be University) Research Kattankulathur, INDIA

## Abstract

**Background:**

Recurrent and metastatic squamous cell carcinoma of the head and neck (R/M SCCHN) presents substantial treatment challenges due to its aggressive and heterogeneous nature. While evidence-based guidelines provide useful recommendations, they may not offer sufficient support for real-time clinical decision-making.

**Objective:**

This Delphi consensus aimed to establish expert agreement on clinical parameters guiding first- and second-line treatment decisions and the management of specific clinical scenarios in R/M SCCHN in France.

**Methods:**

A two-round Delphi consensus was conducted between December 2023 and June 2024. A scientific committee of six national experts developed consensus statements based on a literature review and supervised the process. An expert panel of 25 oncologists from across France participated in both rounds.

**Results:**

All respondents agreed that the need for a rapid therapeutic response and the presence of comorbidities were critical factors influencing first-line treatment choice. Seven major indicators of rapid response were identified: rapidly progressive relapse, partial airway obstruction, respiratory compromise, tumour-related bleeding, uncontrolled symptoms, carotid axis lesions, and pain resistant to third-step analgesics. For patients requiring rapid response, 76% supported using cetuximab with chemotherapy as a first-line option in cases with a combined positive score (CPS) between 1 and 19. Furthermore, 84% recommended immunotherapy (alone or combined with chemotherapy) as first-line treatment for CPS ≥ 20 tumours, while 41% would consider it for CPS 1–19 cases. A strong consensus (80%) held that first-line treatment should not restrict second-line options. Notably, 100% of respondents agreed that all patients should receive immunotherapy, cetuximab, taxanes, and platinum salts within the first two treatment lines.

**Conclusions:**

This Delphi consensus offers valuable, expert-based perspectives on the management of R/M SCCHN in France. It highlights a progressive shift from standardized European guideline frameworks toward a more personalized approach that accounts for individual patient profiles and tumour characteristics. The insights generated provide practical guidance for clinical decision-making and lay the groundwork for future research and optimisation of treatment strategies in real-world settings.

## Introduction

Recurrent and/or metastatic squamous cell carcinoma of the head and neck (R/M SCCHN) is a highly aggressive malignancy with limited therapeutic options. It arises from the squamous cells of the upper aerodigestive tract and is frequently associated with risk factors such as smoking, excessive alcohol consumption, and human papillomavirus (HPV) infection [1]. In 2022, the estimated prevalence of SCCHN in Europe was approximately 650,000 cases, with an incidence ranging from 10 to 15 per 100,000 individuals [2].

Despite significant advances in systemic therapies, the optimal management of R/M SCCHN remains a clinical challenge. This is largely due to the heterogeneity of the disease and the lack of well-defined, personalized treatment guidelines. Moreover, the frailty of many patients with R/M SCCHN often limits the applicability of clinical trial findings to routine clinical practice. Current recommendations from the National Comprehensive Cancer Network (NCCN) [3] and the European Society for Medical Oncology (ESMO) [4] advocate for the use of immune checkpoint inhibitors as first-line therapy, primarily guided by PD-L1 expression levels [3,4]. Anti-programmed cell death ligand 1 (PD-L1) therapies have significantly advanced treatment, particularly in patients with a combined positive score (CPS) ≥20, where they show substantial efficacy [4,5]. For patients with CPS < 1, cetuximab-based chemotherapy remains the gold standard due to its ability to provide rapid disease control [4,6]. In patients with intermediate CPS scores (1–19), treatment decisions are more complex and must consider various clinical parameters, including patient age, tumour location, and the need of rapid therapeutic response [7–9]. While the concept of a “need for rapid response” has been recognized in the literature, precise treatment strategies for such situations remain to be clearly defined [10]. Additionally, real-world clinical decision-making often involves factors that are not explicitly addressed in current guidelines, including symptom burden, comorbidities, nutritional status, and psychosocial considerations. This complexity is further compounded by the limited representation of frail and elderly patients in clinical trials, making it difficult to translate trial evidence into practice for these subgroups [11]. Furthermore, variations in access to therapies, institutional experience, and regional practice patterns can contribute to heterogeneity in treatment approaches across healthcare settings. In such contexts, expert-derived guidance can help bridge evidence gaps and improve consistency in clinical decision-making. Previous studies have highlighted the value of consensus methods such as Delphi techniques in oncology, especially when high-level evidence is lacking or when multiple treatment options are available with similar levels of evidence [12,13]. In this context, expert consensus can provide valuable insight into how oncologists navigate nuanced clinical scenarios and tailor treatments based on patient- and tumour-specific characteristics. Delphi methodology, in particular, is well-suited to consolidate expert opinion in areas where evidence is incomplete or conflicting, and where practice variability is high.

This Delphi consensus aimed to explore therapeutic practices among experienced French oncologists managing R/M SCCHN. The objective was to establish consensus on (i) factors guiding first-line treatment decisions, (ii) criteria defining the need for a rapid therapeutic response, and (iii) optimal treatment sequencing based on patient-specific characteristics. By gathering expert consensus, this study seeks to enhance understanding of clinical decision-making processes and provide practical guidance for the management of R/M SCCHN. The insights generated may also inform future updates to clinical guidelines and support more standardized, yet individualized, approaches to care.

## Materials and methods

A scientific committee comprising six French experts in R/M SCCHN (FC, CE, AD, CB, JF, ES — all authors of this article) oversaw the scientific integrity and ethical standards of the study. The committee identified key topics requiring consensus through a comprehensive literature review, collaboratively designed the questionnaire, analyzed the collected data, and contributed substantially to drafting the final manuscript.

This study employed a Delphi consensus methodology involving medical oncologists from across France. As it did not include patient participation or sensitive personal data, formal ethical approval was not required. Participation was voluntary, and informed consent was obtained electronically. All responses were anonymized and handled confidentially.

### Questionnaire development

The questionnaire was developed following a comprehensive literature review on R/M SCCHN, conducted using databases including PubMed/Medline, Cochrane, Lissa, and Cismef, as well as grey literature sources. Search terms included “squamous cell carcinoma of head and neck” and “treatment of alcoholics and tobacco users,” with filters applied for language (English and French), species (humans), and publication years (2018–2023). A structured algorithm combining relevant keywords and MeSH terms was used (search details available in S1 Table). Clinical situations lacking established consensus were prioritized. Questions focused on first-line treatment criteria, indicators for prioritizing rapid response, and optimal treatment sequences based on patient and tumour features. Factors could be designated as “major” (sufficient alone to justify treatment decisions) or “minor” (relevant but not decisive). The first-round questionnaire included 26 items; the second round, targeting items with no consensus, included 20.

### Expert panel recruitment

A total of 34 oncologists recognized for their expertise in R/M SCCHN were invited via email to participate. Of these, 25 (74%) completed the first-round questionnaire. Selection criteria emphasized peer-reviewed publications, institutional leadership in specialized cancer centers, and geographic diversity across France. Participants were also selected based on their active contribution to local clinical guidelines and scientific societies.

### Delphi process

A two-round Delphi method was employed to reach expert consensus on current R/M SCCHN management in France. This method is a structured, iterative approach that uses multiple rounds of questionnaires and feedback to gather expert opinion, while minimizing group bias. It allows anonymous input from geographically dispersed participants, fostering independent judgment across two voting rounds [14]. It quantifies the level of agreement among experts and identifies both areas of consensus and points of divergence on clinical questions or practices [14–16]. In Round 1, participants completed a questionnaire to express their initial views. In Round 2, statements that had not reached the pre-defined consensus threshold in the first round were aggregated and re-submitted for reassessment, allowing participants to reconsider their responses in light of the group feedback. Notably, the Delphi method attributes equal value to consensus achieved in either round [14]. The two voting rounds took place from March 4^th^ to March 25^th^, 2024, and from April 18^th^ to May 24^th^, 2024.

### Data collection and analysis

The questionnaire was hosted on an secure online platform (https://care-orl.com/), and participants accessed it via personalized links. Weekly reminders encouraged timely completion. Data were collected anonymously and aggregated for analysis. As this study employed the Delphi method, no inferential statistical analyses were conducted. Instead, the methodology relied solely on descriptive statistics, such as agreement rates, to assess the level of consensus. This approach is consistent with standard Delphi methodology, which emphasizes the structured collection of expert opinion over formal hypothesis testing. Consensus was defined as an agreement level of ≥66.7%, in line with previously established criteria [17].

### Ethical considerations

The questionnaire included a statement outlining data protection regulations and participant rights. Informed electronic consent was obtained prior to participation. All responses were anonymous and analyzed only in aggregate. Data collection fully complied with the European Union General Data Protection Regulation (GDPR) [18]. Participants were informed of their rights, including:

- Access, rectification, deletion, restriction, and objection to data processing
- The right to contact the study team for additional information
- The right to file a complaint with the CNIL (Commission Nationale de l’Informatique et des Libertés)

This study did not involve human participants or animals. It was conducted as a Delphi consensus process among expert oncologists in France, without the collection of patient data. As such, no formal ethical approval was required. All participants were fully informed about the purpose of the research and provided voluntary electronic consent before participation. The questionnaire explicitly stated that all responses would remain strictly confidential, anonymous, and analysed only in aggregate form. The data collection and processing were carried out in compliance with French data protection regulations (GDPR – General Data Protection Regulation). Participants were informed of their rights regarding access, rectification, deletion, restriction, and objection to the processing of their data. They were also provided with contact details for further inquiries and were informed that, should they believe their rights were not respected, they could file a complaint with the CNIL.

## Results

### Comprehensive literature review

The literature review identified 766 publications published between January 2018 and December 2023. Of these, 51 were selected for inclusion. An additional six publications from the grey literature were incorporated, resulting in a total of 57 references used to guide questionnaire development following a full-text review. A flowchart detailing the inclusion and exclusion process, adapted from the 2020 PRISMA guidelines [19], is presented in S2 Fig.

### Respondent characteristics

**Fig 1:**
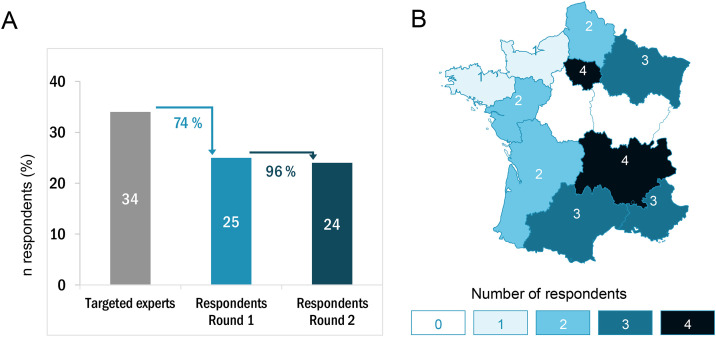
Respondent characteristics. **A.** Sample size including targeted experts and respondents in the first and second rounds. **B.** Geographic distribution of respondents.

Among the 25 experts who participated, all completed the first round of the Delphi process, and 24 (96%) responded in the second round (Fig 1A). All participants were medical oncologists, geographically distributed across France (Fig 1B). The majority (60%) worked in private institutions, including 56% in cancer centers, while 40% were based in university hospitals. The median experience in managing R/M SCCHN was 10 years (range: 3–32), and the median number of new R/M SCCHN patients treated annually was 30 (range: 5–80). The detailed analysis of the data set can be found in S3 Appendix.

### Factors determining first-line treatment

According to respondents, all factors identified through the literature review and included in the questionnaire should be considered when selecting first-line treatment (Table 1). Among these, seven were classified as “major” factors, and one as “minor.” There was no consensus on whether patient age should be considered a major or minor factor, although all respondents agreed it should be taken into account. Notably, the need for a rapid response and the presence of comorbidities were the only factors unanimously (100%) classified as major in determining first-line treatment (Table 1).

**Table 1:** Factors determining the choice of first-line treatment. “*Please classify each factor for selecting the first-line treatment based on whether you perceive them as ‘major’ (i.e., justifying on its own the choice of 1*^*st*^ *line treatment, if present), ‘minor’ (i.e., considered for this choice, but not as a major factor), or ‘not to be taken into consideration.*”.

Proposed factors	Considered (%)	Major (%)	Minor (%)
Tumour needing a rapid response^1^	**100**	**100**	0
Presence of comorbidities^2^	**100**	**100**	0
Combined positive score^1^	**100**	**84**	16
Presence of symptoms^1^	**100**	**83**	17
Performance status^2^	**100**	**76**	24
Time since last platinum salt administration^3^	**100**	**72**	28
Nutritional status^2^	**100**	24	**76**
Patient age^2^	**100**	42	58
Eligibility for cisplatin^3^	**92**	**71**	21

^1^ Factors related to the tumour.

^2^ Factors related to the patient.

^3^ Factors related to treatment.

The values in **bold** are greater than 66.7%; the corresponding factors have been consensually classified.

Note: As required by the Delphi methodology, the data presented are descriptive in nature, and no statistical comparisons were conducted.

### Defining the need for a rapid response

The questionnaire aimed to identify specific indicators of the need for a rapid therapeutic response. Initially, respondents spontaneously cited key factors, with symptomatic disease and pain being the most frequently mentioned (76%), followed by respiratory (64%) and vascular (60%) impairments (Table 2). Subsequently, literature-based factors were presented. Seven of these were consistently classified as major, four as minor, and five did not reach consensus regarding their relevance to rapid response (Table 2).

**Table 2:** Factors used to define a need for a rapid response to the first-line treatment. “*Please classify each factor that, in your opinion, qualify a R/M HNSCC as a ‘tumour requiring a rapid response to first-line treatment,’ based on whether you perceive them as ‘major’ (i.e., justifying a need for a rapid response on its own if present), ‘minor’ (i.e., considered but not as a major factor), or ‘not to be taken into consideration.*”.

Spontaneous factors suggested by expert panel	Percentage consensus, %		
Symptoms and pain	**76**		
Respiratory impairment	64		
Vascular impairment	60		
Speed of evolution	44		
Tumour load or weight	24		
**Proposed factors based on literature review**	**Considered (%)**	**Major (%)**	**Minor (%)**
Rapidly progressive relapse^1^	**100**	**96**	4
Partial airway obstruction^1^	**100**	**92**	8
Respiratory difficulty associated with pulmonary metastatic progression^1^	**100**	**88**	12
Tumour-related bleeding^1^	**100**	**80**	20
Symptoms not controlled by supportive care related to tumour progression, either locoregional or distant^2^	**100**	**76**	24
Presence of pain poorly controlled by third-step analgesics^2^	**100**	**68**	32
Eating difficulties related to tumour recurrence^1^	**100**	63	37
Presence of lesions on a carotid axis^1^	**96**	**72**	24
Hypercalcemia (>3 mM) ^2^	**96**	63	33
Location of metastases exclusively in the liver^3^	**92**	17	**75**
Fast deterioration of general condition over the past month^2^	**88**	50	38
Weight loss ≥5% over the past month ^2^	**84**	16	**68**
Hypoalbuminemia (<30g/L) ^2^	**84**	17	**67**
Asymptomatic liver metastases^1^	**84**	17	**67**
Abnormalities in laboratory tests^2^	**83**	50	33
Presence of more than three metastatic sites^3^	**83**	29	54
Location of metastases exclusively in bones^3^	50	–	–
Asymptomatic local relapse with small to moderate tumour volume^1^	46	–	–
Presence of more than one hepatic lesion^1^	46	–	–
Location of metastases exclusively in the lungs^3^	42	–	–
Asymptomatic bone metastases^1^	44	–	–

^1^ Factors related to the tumour.

^2^ Factors related to the patient (measured in clinical evaluation).

^3^ Proposed only in the second round.

The values in **bold** are greater than 66.7%; the corresponding factors have been consensually classified.

Note: As required by the Delphi methodology, the data presented are descriptive in nature, and no statistical comparisons were conducted.

### Therapeutic management of patients requiring a rapid response

**Fig 2:**
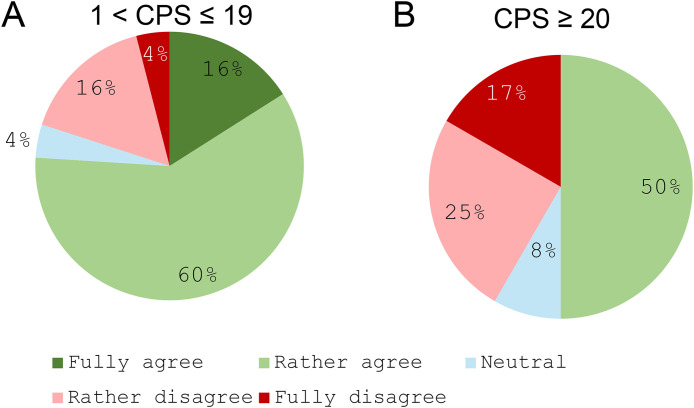
Systematic discussion of the use of a combination of cetuximab with mono- or polychemotherapy as a first-line treatment for patients requiring a rapid response according to the tumour CPS. **A.** Tumours with a CPS between 1 and 19; **B.** Tumours with a CPS ≥ 20. **“For a patient with R/M HNSCC requiring a rapid response, the combination of cetuximab with mono- or polychemotherapy is a therapeutic option that should be systematically considered as a first-line treatment for a CPS score between 1 and 19 for A/ ≥20 for B. Do you: Fully agree/Rather agree/Stay Neutral/Rather disagree/Fully disagree with this statement?”.**

A strong consensus (76%) supported the systematic consideration of cetuximab combined with chemotherapy as a first-line option for patients requiring a rapid response and with a CPS between 1 and 19 (Fig 2A). However, no consensus was reached regarding the systematic discussion of this combination in patients with a CPS ≥ 20 (50% agreement; Fig 2B).

### Use of immunotherapy in first-line treatment

**Fig 3:**
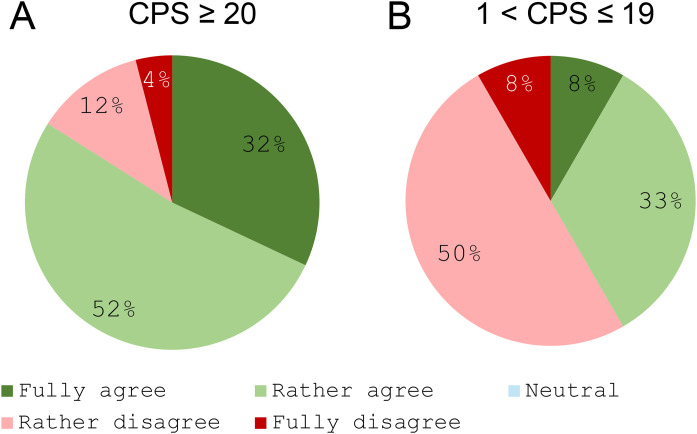
Use of immunotherapy in the first line (alone or in combination with chemotherapy) according to the tumour’s CPS. **A.** Tumours with a CPS ≥ 20; **B.** Tumours with a CPS between 1 and 19. *“A CPS score ≥ 20 for A/ CPS score between 1 and 19 for B in a given patient’s tumor systematically leads to the prescription of immunotherapy (alone or in combination with chemotherapy) as a first-line treatment, regardless of the patient’s clinical situation (except in cases of contraindication to immunotherapy). Do you: Fully agree/Rather agree/Stay Neutral/Rather disagree/Fully disagree with this statement?”.*

There was strong consensus among respondents on the use of immunotherapy as a first-line treatment in patients with a high CPS. Specifically, 84% agreed that a tumour with CPS ≥ 20 should automatically prompt consideration of immunotherapy, either alone or combined with chemotherapy, in the first-line setting (Fig 3A). Conversely, only 41% supported routine use of immunotherapy for patients with CPS scores between 1 and 19, indicating a lack of consensus for this group (Fig 3B).

Respondents reported a median of 24% (range: 5–90%) of patients with CPS ≥ 1 receiving immunotherapy alone as first-line treatment. All respondents (100%) agreed that immunotherapy alone should primarily be considered for patients with CPS ≥ 20 who do not require a rapid response.

**Fig 4:**
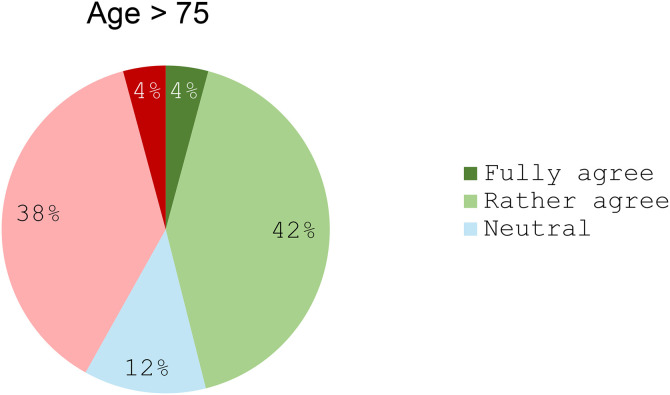
Use of immunotherapy alone for elderly persons (>75 years old). “First-line immunotherapy alone should be preferred for elderly patients. Do you: Fully agree/Rather agree/Stay Neutral/Rather disagree/Fully disagree with this statement?”.

No consensus was reached on the use of immunotherapy alone in patients over 75 years of age, with only 46% supporting its use (Fig 4). Most respondents (92%) emphasized the need for close clinical monitoring in patients treated with immunotherapy alone. Regarding radiologic follow-up, responses indicated a median monitoring interval of 8 weeks (range: 6–16 weeks) for patients at risk of rapid progression, and 12 weeks (range: 6–16 weeks) for those not at risk (Table 3).

**Table 3:** Radiologic surveillance: time interval between radiologic examinations for patients with R/M SCCHN treated by immunotherapy alone, according to the risk of rapid progression. *“What frequency of radiological monitoring would you recommend for patients treated with immunotherapy in order to identify potential progression and switch to second-line treatment as soon as necessary?”.*

Risk of rapid progression^1^	Yes	No
**Median, weeks**	8	12
**Range (minimum; maximum)**	(6; 16)	(6; 16)

^1^ Progression of at least 20% of the tumour in the short term.

Note: As required by the Delphi methodology, the data presented are descriptive in nature, and no statistical comparisons were conducted.

### Therapeutic sequence

**Fig 5:**
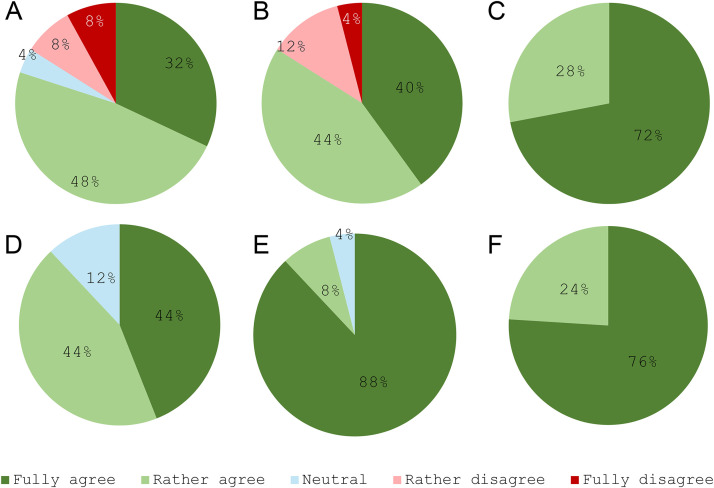
Consensus on the therapeutic sequence in managing R/M SCCHN. **A.** First-line treatment choice should not hinder second-line options; **B.** Therapeutic management should follow the best therapeutic sequence; **C.** Patients should receive immunotherapy, cetuximab, taxanes, and platinum salts within the first two treatment lines; **D.** Cetuximab and taxanes should be considered as second-line treatment after progression with immunotherapy and chemotherapy; **E.** The combination of cetuximab and chemotherapy should be considered after progression with immunotherapy alone; **F.** Immunotherapy should be considered after progression with cetuximab and chemotherapy, even if CPS < 1. *“Do you: Fully agree/Rather agree/Stay Neutral/Rather disagree/Fully disagree with A, B, C, D, E, F statements?”.*

Consensus was achieved on several aspects of treatment sequencing. Overall, 80% of respondents agreed that the choice of first-line therapy should not compromise the availability of second-line options (Fig 5A). Furthermore, 84% highlighted the importance of basing R/M SCCHN management on the most effective therapeutic sequence (Fig 5B). Notably, all respondents (100%) agreed that every patient should receive immunotherapy, cetuximab, taxanes, and platinum salts within the first two lines of treatment (Fig 5C).

When immunotherapy combined with chemotherapy was used as first-line treatment, 88% of respondents supported systematic consideration of cetuximab with taxanes as a second-line option (Fig 5D). When immunotherapy alone was used in the first line, 96% agreed systematically discussing cetuximab with mono- or polychemotherapy in the second line (Fig 5E). In cases where first-line treatment included cetuximab combined with chemotherapy, 100% of respondents agreed that immunotherapy should be systematically discussed as a second-line option, even for patients with CPS < 1 (Fig 5F). Finally, 96% of respondents noted that the flexible administration of cetuximab makes it highly adaptable to the individual characteristics and clinical needs of most patients.

## Discussion

This Delphi consensus study was designed to investigate clinical practices among French medical oncologists managing R/M SCCHN and to reach expert agreement on key clinical scenarios where practical guidance is currently lacking. These include identifying relevant factors influencing first-line treatment decisions, establishing criteria to define urgency for therapeutic response, and optimizing treatment sequencing based on patient-specific factors.

A total of 34 medical oncologists with substantial experience in R/M SCCHN were invited to participate in the Delphi process, with a high response rate: 74% completed the first-round questionnaire, and 96% of those completed the second round. The final panel of 25 oncologists had a median of 10 years of clinical experience treating R/M SCCHN and managed a median of 30 new patients per year. Notably, most respondents (56%) practiced in cancer centres and 40% in large public hospitals, indicating a high level of expertise and exposure to complex clinical scenarios. However, this distribution may under-represent perspectives from smaller or rural settings. The expert panel consisted exclusively of medical oncologists, selected for their advanced expertise in systemic therapies, including chemotherapy and immunotherapy, thereby ensuring the relevance and consistency of the responses. They were chosen based on their substantial research involvement and academic contributions to scientific publications, ensuring that the insights collected reflected the perspectives of recognized experts with up-to-date knowledge of recent developments in the management of R/M SCCHN.

In terms of sample size, Delphi methodology has no formal requirements [15], a review of previous studies shows that sample sizes ranged from 3 to 418 participants, with a median of 17 being commonly reported [20]. The sample size of 25 French participating medical oncologists is considered appropriate and consistent with previous Delphi studies in oncology. For comparison, a Spanish Delphi consensus included 25 specialists across medical and radiation oncology [21], while an Italian Delphi study recently involved a multidisciplinary panel of 39 experts [22]. Given the rarity and complexity of R/M SCCHN, which is typically managed in referral centres, the number of medical oncologists with advanced expertise in France is relatively limited. Thus, our panel size represents a substantial and nationally representative sample, with geographical diversity and a range of institutional backgrounds. This ensures a reliable and comprehensive perspective on current national practices. Future research could benefit from the inclusion of other specialties, such as radiotherapists and head and neck surgeons, to broaden the scope.

The panel of respondents reached consensus on six key criteria as major determinants in selecting first-line treatment: the “need for rapid response” (100%), the “presence of comorbidities” (100%), CPS (84%), the “presence of symptoms” (83%), “performance status” (76%), and “time since the last platinum-based chemotherapy” (72%). Although all respondents agreed that patient age should be considered, there was no consensus on whether it should be categorized as a major or minor factor. The scientific committee highlighted the need for multidisciplinary discussion and the integration of additional clinical factors beyond patient age. The role of comorbidities was particularly emphasized. In the literature, specific conditions such as significant cardiovascular disease, renal impairment, hepatic dysfunction and autoimmune disorders were noted to limit the use of immunotherapy or cytotoxic agents [23–27]. In such cases, less toxic regimens such as PCC (paclitaxel, carboplatin, cetuximab) are considered more appropriate. These observations align with real-world data, including the DIRECT study [28], which evaluated cetuximab-based therapies in patients with comorbidities. The findings reinforce the need to individualize treatment decisions based on both patient characteristics and disease presentation, particularly in scenarios requiring a rapid therapeutic response.

The role of CPS in treatment selection was also recognized, especially for stratifying patients based on PD-L1 expression. However, the panel strongly supported the integration of clinical urgency with CPS to guide therapeutic choice. In stable patients with CPS ≥ 20, immunotherapy monotherapy was favored, while in patients with symptomatic or rapidly progressing disease, cetuximab-based combinations were considered more appropriate regardless of CPS. This approach reflects an evolution from biomarker-exclusive models to individualized strategies incorporating clinical dynamics, a perspective echoed in studies focusing on the same tumour type (R/M SCCHN) including Peyrade et al. (2021) [7], Bossi et al. (2025) [22], and Simon et al. (2025) [29].

Notably, the Delphi consensus highlighted a significant gap between algorithmic guidelines and nuanced real-world practice. While ESMO guidelines [4] prioritize CPS as the principal decision-making criterion, our findings are more closely aligned with the NCCN approach [3], which allows for clinical discretion in complex cases. However, the NCCN guidelines, while advocating for the consideration of various clinical and patient-specific factors, do not offer precise recommendations on specific management strategies. The panel emphasized the importance of tailoring treatment decisions to each patient’s clinical presentation, frailty status, and disease kinetics. Expanding on this perspective, *Peyrade et al., 2021* offered more detailed recommendations for clinical decision-making, introducing the “need for a rapid response” as a key criterion, alongside CPS and overall patient health, in guiding the choice of first-line treatment [7].

The Delphi panel defined seven key clinical indicators that define the urgency of therapeutic response: rapidly progressive relapse (96%), partial airway obstruction (92%), respiratory issues due to pulmonary metastases (88%), tumour-related bleeding (80%), uncontrolled symptoms (76%), refractory pain (68%), and carotid axis impairment (72%). These markers were selected for their association with immediate clinical risk and symptom burden. These insights build upon prior expert commentary [7] and are consistent with prognostic frameworks proposed by *Szturz et al. (2025)* [30] and Yang et al. (2024) [31]. Other conditions, such as isolated liver metastases, hypoalbuminemia, recent weight loss, and asymptomatic lesions, were seen as minor or context-dependent indicators of urgency. Although insufficient alone to justify a rapid response, their presence may reflect a more aggressive disease course when combined. This suggests the importance of incorporating these elements into the overall clinical decision-making process. Additional factors including eating difficulties related to recurrence, hypercalcemia, rapid deterioration of general condition, biological abnormalities, and a high number of metastatic sites were mentioned but did not reach consensus, as respondents were divided in how they classified their clinical relevance. Similarly, factors such as isolated bone or lung metastases, asymptomatic local relapse with low tumour volume, multiple liver lesions, and non-symptomatic bone metastases did not reach consensus regarding their role in determining the need for a rapid therapeutic response. In the literature, urgent treatment is typically recommended in cases of high tumour burden, characterized by rapid disease progression, compressive symptoms, or severe pain [4,32–34]. Several authors have described these tumours as “aggressive” and “rapidly progressive,” highlighting their potential to be life-threatening or to severely compromise vital functions [7,9,35]. Our findings contribute to refining existing definitions in the literature, particularly in cases of fast-growing disease requiring “an enhanced response rate” [32] or scenarios where “rapid tumour shrinkage is needed” [4].

The present findings, conducted after several years of clinical experience with key therapeutic options, confirm the importance of certain combination regimens in the management of R/M SCCHN. Notably, 76% of participants agreed that cetuximab–chemotherapy combinations should be systematically considered as a first-line treatment for patients with a CPS between 1 and 19 when a rapid response is needed. This is consistent with expert guidance previously outlined by *Peyrade et al*., *2021* [7] and reinforced by *Libert et al.* (2025) [36], whose retrospective analysis of the TPEx regimen (cetuximab plus platinum salts and docetaxel) demonstrated robust response rates and tolerability, particularly in fit patients. Although immunotherapy combined with chemotherapy was also mentioned as a possible alternative, TPEx was favored in this setting due to its high response rate (approximately 55%) and disease control rate (around 80%), along with encouraging median overall survival outcomes [37]. One hypothesis explaining this preference is the rapid tumour shrinkage observed with TPEx, which makes it especially suitable for patients in urgent need of disease control.

Conversely, no consensus was reached regarding the need to systematically discuss cetuximab–chemotherapy combinations as a first-line option for patients with CPS ≥ 20 requiring a rapid response, a context in which immunotherapy is generally more effective. This aligns with the high level of agreement (84%) that immunotherapy should be systematically used as first-line treatment for patients with CPS ≥ 20, in contrast to the lower agreement (41%) for its use in those with CPS between 1 and 19. These views are consistent with updated findings from the KEYNOTE-048 study [38] and further supported by Simon et al. (2025) [29], who demonstrated superior real-world outcomes of IO in CPS ≥ 20 but more heterogeneous results in intermediate CPS scores.

The responding panel also discussed the role of immunotherapy monotherapy as a first-line treatment for R/M SCCHN. On average, respondents reported that 24% of their patients with a CPS > 1 received immunotherapy alone in the first-line setting, with substantial variation across centres (ranging from 5% to 90%). This average was considered realistic by the scientific committee, although values exceeding 50% were viewed as unusually high. These discrepancies likely reflect differences in patient demographics, interpretations of the need for a rapid response definition, capacity for close monitoring, and local clinical practices across centres. Importantly, unanimous consensus (100%) was reached in support of using immunotherapy alone in patients with a CPS ≥ 20 who do not require a rapid therapeutic response. However, no consensus emerged regarding its systematic use in patients with a CPS between 1 and 19, or in elderly patients (generally defined as over 75 years). Support for immunotherapy monotherapy is largely based on its favorable toxicity profile and the survival benefits observed in both the KEYNOTE-048 trial and real-world evidence [29].

Nonetheless, particular caution is warranted given the potential risk of rapid disease progression associated with immunotherapy monotherapy [39,40]. Its use should be limited to patients whose disease does not necessitate an urgent response, even when CPS is high. While the ESMO guidelines do not specify monitoring frequency for patients receiving immunotherapy alone, they implicitly suggest the importance of close follow-up to detect early progression and adapt treatment accordingly [4]. This need was echoed in our consensus, with 92% of respondents affirming the importance of close clinical monitoring for patients receiving immunotherapy monotherapy. Immunotherapy monotherapy is biologically justified by its mechanism of action, which involves stimulating the host immune system to target PD-L1–expressing tumours. Clinically, it offers a well-tolerated alternative to combination regimens for patients without a pressing disease burden, providing therapeutic benefit without adding the toxicity associated with chemotherapy. The KEYNOTE-048 trial [38] along with real-world evidence such as the study by Simon et al. (2025) [29], demonstrated significant survival benefits, particularly in patients with a CPS ≥ 20. However, panelists also acknowledged the potential risk of hyperprogression, as highlighted by *Saâda-Bouzid et al.* (2017) [39], underscoring the importance of careful patient selection and close clinical monitoring. This divergence in opinion reflects the complexity of therapeutic decision-making and reinforces the need for individualized treatment strategies tailored to each patient’s clinical and biological profile.

Therapeutic sequencing emerged as another area of consensus. A strong majority (84%) emphasized the importance of guiding R/M SCCHN treatment based on the most effective sequence. All respondents (100%) agreed that each patient should have access to four key agents: immunotherapy, cetuximab, taxanes, and platinum salts within the first two treatment lines. When first-line treatment involved immunotherapy combined with chemotherapy, there was strong consensus (88%) that the cetuximab–taxane combination should be systematically discussed as a second-line option. Although off-label in this setting, this regimen has been investigated in several studies for patients with R/M SCCHN following relapse after immunotherapy, demonstrating encouraging results in terms of response rate, disease control, and overall survival, with an acceptable benefit–risk profile for this population [41–48]. Additionally, 96% endorsed cetuximab-based regimens after immunotherapy monotherapy. Conversely, all respondents (100%) agreed that immunotherapy should be considered as a second-line treatment following a first-line regimen of cetuximab and chemotherapy, even in patients with tumours exhibiting a CPS < 1. This reflects emerging evidence supporting cross-line efficacy [30,34] and the feasibility of reintroducing immunotherapy in selected contexts. It underscores the importance of treatment sequencing strategies that consider prior regimens and patient response, rather than relying exclusively on biomarker thresholds.

Moreover, the panel acknowledged the clinical value of cetuximab-based regimens, especially in frail or elderly patients. Most participants (96%) highlighted the flexibility of cetuximab administration, which facilitates treatment individualization for frail patients with reduced physiological reserve or contraindications to standard therapies. One notable example is the PCC regimen (paclitaxel, carboplatin, cetuximab), which delivers reduced doses of paclitaxel and carboplatin on a weekly basis alongside cetuximab in the first-line setting. This regimen has shown promising efficacy including overall survival benefits in frail patients, with a manageable toxicity profile [29,49]. These findings highlight the importance of adaptable treatment strategies for patients who are less able to tolerate conventional protocols.

In summary, this consensus provides meaningful and actionable guidance for both clinical practice and the design of future trials in R/M SCCHN. By defining the concept of a “tumour requiring a rapid response” and identifying key stratification factors, such as urgency of response, comorbidities, frailty indicators, and real-world eligibility, our findings support more personalized and clinically relevant treatment strategies. Stratifying patients based on these factors enables clinicians to allocate resources more effectively, distinguishing those who require urgent intervention from those who may be better suited to less intensive monitoring or therapy. Furthermore, the proposed criteria have the potential to strengthen the external validity of future clinical trials by improving patient selection and promoting the use of composite endpoints—such as symptom relief, early disease control, and quality of life—that more accurately reflect real-world outcomes. While differences in clinical judgment may pose implementation challenges, this study highlights the need for practical guidelines, clinician education, and decision-support tools to ensure consistent application across care settings.

Ultimately, our findings provide a solid framework for guiding treatment sequencing and optimizing long-term outcomes in head and neck cancers beyond the first-line setting.

## Conclusions

This Delphi-based consensus provides valuable expert insights into clinical decision-making and practical strategies for managing R/M SCCHN. Consensus was reached on critical factors guiding the selection of first-line treatments and on defining clinical scenarios that warrant a rapid therapeutic response. The findings highlight the importance of tailoring treatment strategies to both patient and tumour characteristics, with particular emphasis on the urgency of intervention and the role of CPS levels. Moreover, the results underscore the increasing relevance of therapeutic sequencing beyond first-line treatment. There was strong agreement on the need to ensure that all patients have access to the four key therapeutic agents (immunotherapy, platinum salts, taxanes, and cetuximab) within the first two lines of therapy. Importantly, the consensus reflects an evolution in clinical practice toward a more individualized and context-driven approach, distinguishing it from current European guideline frameworks. These expert-driven insights form a robust foundation for optimizing patient care and shaping future research in the management of R/M SCCHN.

## Supporting information

S1 Table Literature search algorithm. (DOCX)

S2 Fig Literature search flowchart. (DOCX)

S3 Appendix Data set. (XLSX)
