# Correction: Defining a need for rapid response and practical guidance for recurrent and metastatic squamous cell carcinoma of the head and neck (R/M SCCHN) management in France: A Delphi consensus

**DOI:** 10.1371/journal.pone.0339448

**Published:** 2025-12-22

**Authors:** The PLOS One Staff

The Acknowledgement section of this article is not indicated. The Acknowledgement section is: The authors thank all the participants in this Delphi-based consensus (Dr. Cyril Abdeddaim, Dr. Matthieu Bainaud, Dr. Maureen Bernadach, Dr. Amandine Bruyas, Dr. Sophie Duplomb, Dr. Lionnel Geoffrois, Dr. Aline Houessinon, Dr. Léa Loriguet, Dr. Carole Pflumio, Dr. Coralie Prebet, Dr. Frédéric Rolland, Prof. Sébastien Salas, Dr. Victor Sarradin, Dr. Clémence Toullec, Dr. Julie Vanbockstael, Dr. Marie Vinches, and Dr. Aurore Vozy) and Public Health Expertise for the survey conduct and medical writing support.

There are errors in the Author Contributions. The correct contributions are:

**Conceptualization:** Florian Clatot, Caroline Even, Amaury Daste, Christian Borel, Jérôme Fayette, Myriam Chebbah, Solenn Le Clanche, Esma Saada

**Formal analysis:** Myriam Chebbah, Solenn Le Clanche

**Resources:** Myriam Chebbah, Solenn Le Clanche

**Validation:** Florian Clatot, Caroline Even, Amaury Daste, Christian Borel, Jérôme Fayette, Esma Saada

**Writing – original draft:** Myriam Chebbah, Solenn Le Clanche

**Writing – review & editing**: Florian Clatot, Caroline Even, Amaury Daste, Christian Borel, Jérôme Fayette, Myriam Chebbah, Solenn Le Clanche, Esma Saada

The publisher apologizes for these errors.
