# Supplementary material for: Defining a need for rapid response and practical guidance for recurrent and metastatic squamous cell carcinoma of the head and neck (R/M SCCHN) management in France: A Delphi consensus
Source: PLoS One. 2025 Sep 19;20(9):e0332413. doi: 10.1371/journal.pone.0332413 (PMC12448362; doi:10.1371/journal.pone.0332413)
Supplement: S1 Table — (DOCX) [file pone.0332413.s001.docx]

**Supporting information**

**S1 Table. Literature search algorithm**

| **N°** | **Search Algorithms** | **Results** | **Selected articles*** |
| --- | --- | --- | --- |
| **SCCHN and Guidelines** | | | |
| #1 | ((("head and neck"[Title] OR "oral cavity"[Title] OR larynx[Title] OR oropharynx[Title] OR hypopharynx[Title) AND "squamous cell carcinoma"[Title]) OR HNSCC[Title]) AND (guideline*[Title] OR consensus[Title] OR recommendation*[Title])  Filters: Language: English/French Period: 2018-2023 | 263 | 4 |
| **SCCHN and Therapeutic sequence** | | | |
| #2 | (((("head and neck"[Title/Abstract] OR "oral cavity"[Title/Abstract] OR larynx[Title/Abstract] OR oropharynx[Title/Abstract] OR hypopharynx[Title/Abstract]) AND "squamous cell carcinoma"[Title/Abstract]) OR HNSCC[Title/Abstract]) AND (sequence*[Title/Abstract] OR subsequent[Title/Abstract]))  Filters: Language: English/French Period: 2018-2023 | 109 | 12 |
| **SCCHN and Threatening disease** | | | |
| #3 | ((("head and neck"[Title/Abstract] OR "oral cavity"[Title/Abstract] OR larynx[Title/Abstract] OR oropharynx[Title/Abstract] OR hypopharynx[Title/Abstract]) AND "squamous cell carcinoma"[Title/Abstract]) OR HNSCC[Title/Abstract]) AND (recurrent[Title/Abstract] OR metastatic[Title/Abstract]) AND (threatening[Title/Abstract] OR aggressive[Title/Abstract] OR rapid[Title/Abstract] OR fast[Title/Abstract] OR expanding[Title/Abstract] OR growing[Title/Abstract] OR hyperprogression[Title/Abstract] OR hyperprogressive[Title/Abstract])  Filters: Language: English/French Period: 2018-2023 | 187 | 4 |
| **SCCHN and therapies** | | | |
| #4 | ((("head and neck"[Title] OR "oral cavity"[Title] OR larynx[Title] OR oropharynx[Title] OR hypopharynx[Title]) AND "squamous cell carcinoma"[Title]) OR HNSCC[Title]) AND (recurrent[Title] OR metastatic[Title) AND (management[Title] OR managing[Title] OR treatment[Title] OR treating[Title] OR pembrolizumab[Title] OR nivolumab[Title] OR ipilimumab[Title] OR durvalumab[Title] OR cetuximab[Title] OR chemotherapy[Title] OR docetaxel[Title] OR paclitaxel[Title] OR platinum[Title] OR cisplatin[Title] OR carboplatin[Title])  Filters: Language: English/French Period: 2018-2023 | 207 | 31 |
| **TOTAL** | | **766** | **51** |

*Exclusion criteria included out of scope, duplicate, full-text unavailable.
