# Supplementary material for: Defining a need for rapid response and practical guidance for recurrent and metastatic squamous cell carcinoma of the head and neck (R/M SCCHN) management in France: A Delphi consensus
Source: PLoS One. 2025 Sep 19;20(9):e0332413. doi: 10.1371/journal.pone.0332413 (PMC12448362; doi:10.1371/journal.pone.0332413)
Supplement: S2 Fig — (DOCX) [file pone.0332413.s002.docx]

**Supporting information**

**S2 Figure. Literature search flowchart**

**Selection**

*Publications excluded
(n = 715)*

*Reasons of exclusion:*

- *Not the data of interest (n = 672)*
- *Duplicate (n = 29)*
- *Not available (n = 12)*
- *Wrong language (n = 2)*

**Included publications**

**(n = 51)**

**Algorithm**

Total publications

(n = 766)

*Grey literature
(n = 6)*

***TOTAL
(n = 57)***
